# Spinal Ependymomas: An Updated WHO Classification and a Narrative Review

**DOI:** 10.7759/cureus.49086

**Published:** 2023-11-20

**Authors:** Eliezer Villanueva-Castro, Juan Marcos Meraz-Soto, Itzel Ariadna Hernández-Dehesa, Martha Lilia Tena-Suck, Rebeca Hernández-Reséndiz, Edgardo de Jesus Mateo-Nouel, Juan Antonio Ponce-Gómez, Juan Nicasio Arriada-Mendicoa

**Affiliations:** 1 Department of Neurosurgery, Instituto Nacional de Neurología y Neurocirugía Manuel Velasco Suárez, Mexico City, MEX; 2 Department of Neuroimaging, Instituto Nacional de Neurología y Neurocirugía Manuel Velasco Suárez, Mexico City, MEX; 3 Department of Neuropathology, Instituto Nacional de Neurología y Neurocirugía Manuel Velasco Suárez, Mexico City, MEX; 4 Department of Pediatric Neurology, Hospital Angeles Universidad, Mexico City, MEX

**Keywords:** neuro-oncology surgery, ependymomas, literature review, central nervous system tumors, spinal ependymoma

## Abstract

Ependymomas are neuroepithelial tumors that develop from ependymal cells found in the brain parenchyma and can spread to any part of the spinal cord. Three to six percent of all malignancies affecting the central nervous system (CNS) are ependymomas. Even the most talented surgeons are challenged by spinal cord ependymomas; as a result, research into this clinical phenomenon should continue. Since 1979, the World Health Organization (WHO) has published a classification and grading system for CNS malignancies to ensure consistent diagnostic standards worldwide. The WHO prepared an update on these tumors, paying particular attention to molecular techniques to categorize the therapeutic management of each patient with greater accuracy and clarity. We thoroughly reviewed the literature on the epidemiology, etiology, diagnosis, and treatment of spinal ependymomas since there has not been a recent review of these tumors. This included modifications to the 2021 WHO Classification of Tumors of the Central Nervous System.

## Introduction and background

Percival Bailey reported the first comprehensive study of ependymomas in 1924. Our knowledge of ependymomas has grown exponentially since then, chiefly in the last few years [1]. The World Health Organization (WHO) has released a categorization and grading of central nervous system (CNS) tumors, aiming to guarantee consistent diagnostic criteria worldwide since 1979. In the most recent CNS edition, the WHO created an update on these tumors, giving specific attention to molecular approaches to categorize the therapeutic management of each patient with more accuracy and to have more clarity on the prognosis [2]. Neuroimaging techniques, particularly magnetic resonance imaging (MRI), provide crucial information regarding tumor features, enabling better planning before surgery and improving molecular techniques [3]. Even with all these developments, spinal cord ependymomas continue challenging even the most skilled surgeons; consequently, research into this clinical entity should continue progressing. Since 2020, methylome profiling findings have shown that molecular groups of ependymal tumors in the posterior fossa, supratentorial, and spinal compartments are distinct, prompting the Consortium to Inform Molecular and Practical Approaches to CNS Tumor Taxonomy - Not Official WHO (cIMPACT-NOW) group to insist on update 7; separating ependymal tumors by anatomic site is a key principle of the new classification [4]. Despite this, there has been no recent review of spinal ependymomas (SP-EPs); hence, we conducted a narrative review of the literature on the epidemiology, etiology, diagnosis, and treatment of these tumors, including changes in the 2021 WHO Classification of Tumors of the Central Nervous System.

Objective

In our narrative review, we aimed to comprehensively review the epidemiology, etiology, diagnosis, and treatment of spinal ependymomas. Our analysis incorporates the latest revisions from the 2021 WHO Classification of Tumors of the Central Nervous System, providing current data to inform optimal management strategies.

Methods

We performed a literature review using the databases PubMed, Google Scholar, and Scopus. A search was conducted for English articles published between 2013 and 2023. The search terms were spinal ependymomas, spinal cord ependymomas and intradural spinal tumors. Three reviewers independently evaluated each stage of the article selection process, and a manual search of relevant references was conducted.

**Figure 1:**
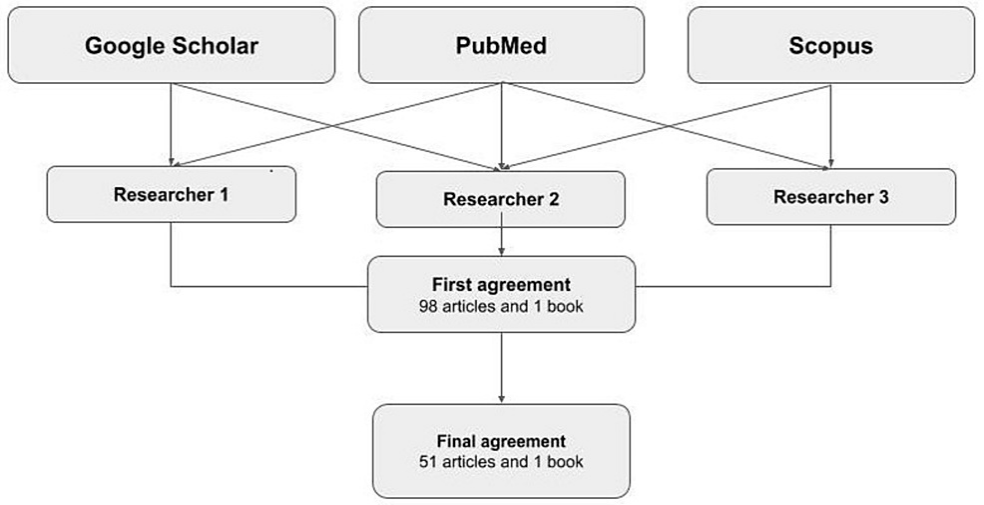
Search strategy

The initial search yielded 1,766 references, and according to the search engine, the results were organized by relevance. Three researchers carefully read the titles and abstracts of the first 500 articles in both databases. During the initial agreement, 46 articles from PubMed, 38 from Google Scholar, and 14 from Scopus were included. Articles with non-English full texts, articles that were not found in the search, articles with content that did not match the purpose of our research, and duplicate articles were excluded. Finally, 27 PubMed articles, 16 Google Scholar articles, 8 Scopus articles, and a book corresponding to the WHO classification of CNS tumors issued in 2021 were included (Figure 1).

## Review

Definition

Ependymomas are neuroepithelial tumors arising from ependymal cells throughout the whole neuroaxis, from the brain parenchyma to any level of the spinal cord, the latter being the most prevalent location in adults. Ependymal cells are fundamental for cerebrospinal fluid (CSF) production [5-7].

Epidemiology

Ependymomas compose 3%-6% of all CNS tumors, of which almost 50% come from the spinal cord [8,9]. Primary spinal cord tumors are uncommon, accounting for 4%-10% of all CNS tumors; however, ependymomas remain the most common primary intramedullary spinal cord tumors, representing 30%-45% of such lesions [8,10].

According to the Central Brain Tumor Registry of the United States (CBTRUS), the annual incidence of all ependymomas is higher in males than females and ranges from 0.29 to 0.60 per 100,000 people [9]. Incidence rates by race are more elevated in whites than in blacks [9,10]. Spinal cord tumors account for a relatively small percentage of primary brain and other CNS tumors; nevertheless, the predominant histology group for those aged 0-19 years was ependymal tumors (19.6%)[9]. Statistically significant differences in rates by histology type between sexes were found for spinal cord ependymomas (male:female rate ratio, 1.15) [8,9]. Across various studies, the median age at diagnosis of patients with SP-EP ranges from 25 to 45 years [10]. Ependymomas differ significantly between pediatric and adult populations. These tumors account for about 5% of all CNS tumors in adults and 10% in pediatric cases. Interestingly, the anatomical distribution varies significantly, with nearly half of the adult ependymomas arising in the spinal cord, compared to almost 90% of these tumors in the pediatric population [11].

Etiology and pathogenesis

SP-EPs are more common in individuals with neurofibromatosis type 2 who have germline nonsense and frameshift mutations in the neurofibromatosis type 2 (NF2) gene than in patients with other types of NF2 mutations [12]. The SP-EP frequently has chromosome 22q loss, although no specific tumor suppressor genes in this region are consistently altered [13]. Despite its location on chromosome 22q, there is no evidence that merlin (NF2) contributes to the pathogenesis of SP-EP [13,14]. SP-EPs affect 18%-53% of patients with neurofibromatosis type 2, with clinical symptoms appearing in less than 20% of cases [15]; 39% of SP-EP patients had NF2 insertion/deletion or nonsense mutations [12].

Myxopapillary ependymomas (MPEs) have the most genetic abnormalities of any SP-EP subtype, with mutations in chromosome 7 being the most common [13,16].

Immunolabeling methods have investigated mutations linked to SP-EP tumorigenesis and prognostic markers. Although p53 mutations are uncommon in SP-EP, the MDM2 gene is amplified and overexpressed, which regulates p53-mediated cell growth [13]. Furthermore, although the difference is not statistically significant, p53 expression may be higher in grade II and III ependymomas than in subependymomas (SEs) [13]. Ki-67, a nuclear protein linked to proliferation, increased expression from grade I to grade III SP-EP, suggesting that it could be employed as a prognostic marker.

In addition, the ErbB protein family has been studied as a therapeutic target in SP-EPs [13,16]. Co-expression of ErbB 2 (Her2) and ErbB 4 has been identified in over 75% of ependymomas, and ligand-dependent activation of the ErbB receptor induces cellular proliferation in cultured ependymoma cells. This proliferation signaling cascade was then inhibited in a dose-dependent manner by a novel ErbB 2 tyrosine kinase inhibitor [13,16]. Furthermore, the inhibition of ependymal cell growth after treatment with a topoisomerase inhibitor (WP744), as well as p-STAT3/hypoxia-inducible factor 1α (HIF-a) inhibitors (WP1066 and WP1193), potentially indicates additional molecular therapeutic targets in SP-EPs [17].

Current 2021 WHO CNS tumor classification: a spinal ependymoma context

Since 1979, the WHO has developed a classification of CNS tumors to standardize globally applicable diagnostic criteria (Table 1) [18,19]. Traditionally, the WHO has quantitatively stratified CNS tumors, specifically discussing SP-EPs, based on their histological features. The 2016 CNS tumor classification categorized SP-EPs as SE (WHO grade 1), MPE (WHO grade 1), the classic ependymoma with its three histological subtypes, papillary, clear cell, and tanycytic (WHO grade 2), anaplastic ependymoma (WHO grade 3) and one genetically defined type of ependymoma, RELA fusion‐positive (WHO grade 2/3) [20]. However, the association between tumor grade and patient clinical outcome is limited, and its usefulness has been subject to discussion [20]. To overcome these limitations, the current classification focused on developing molecular profiles using various techniques, particularly DNA methylation, which is a significant adjunct for tumor diagnosis [18,21].

**Table 1:** Comparison of key features of and diagnostic criteria for spinal ependymomas between 2016 and 2021 WHO CNS tumor classifications A check mark indicates a very low progression rate and a good overall survival, while a cross sign indicates a high progression rate and a poor survival prognosis. The morphological and immunohistological properties of ependymomas are necessary criteria for all ependymoma types' diagnosis. CNS, central nervous system; SP-EP, spinal ependymoma; SP-MYCN, spinal ependymoma MYCN-amplified; SP-MPE, spinal myxopapillary ependymoma; SP-SE, spinal subependymoma; SP-EPN, spinal ependymoma (anaplastic).

	Age group	Grade	Molecular features	Diagnosis criteria	Prognosis
WHO CNS tumor classification (4th edition, 2016)					
SP-SE	Adults	I	—	Thoracic localization	☑
SP-MPE	Adults	I	—	Lumbar localization	☑
SP-EPN	Adults	II/III	—	Thoracic-lumbar localization, high cell density, elevated mitotic count alongside widespread microvascular proliferation and palisaded necrosis	☒
WHO CNS tumor classification (5th edition, 2021)					
SP-SE	Adults	1	TERT mutations, Chr 6 deletion	Thoracic localization, circumscribed glioma, clustering of tumor cell nucleoli in expansive, no conspicuous nuclear atypia, absent or minimal mitotic activity, DNA methylation class SP-SE (unresolved)	☑
SP-EP	Adults	2/3	NF 2 mutation, Chr 22q deletion	Spinal localization and absence of morphological features of SP-MP or SP-SE	☑
SP-MPE	Adults	2	Chr instability	Filum terminale or conus medullaris localization, papillary structures and/or perivascular myxoid change, immunoreactivity for GFAP, methylation class MP (unresolved)	☑
SP-MYCN	Adults	—	MYCN amplification (Chr 2p)	Cervical or thoracic localization, MYCN amplification	☒

2021 WHO CNS tumor classification main modifications

Ependymoma Definition

Morphological and immunohistopathological characteristics of ependymal tumors are required to diagnose all ependymomas. The distinct tumor types are defined by their location in one of the neuroanatomical compartments and by particular molecular and, in some instances, immunohistochemical criteria [21]. SEs and MPEs are exceptions in that no specific anatomical location is required. SEs can arise in any of the different anatomical regions. The great majority of MPEs are located in the lower segments of the spinal cord; only a few cases to have developed cranially or even outside of the CNS have been reported, albeit the bulk of these have not been validated on a molecular level.

Usage of the Term

Arabic numerals have replaced Roman numerals in the grading system to assist in distinguishing it from the previous classification; the keyword "type" has replaced "entity," and the term "subtype" has become "variant" [18].

Grading

The ependymal neoplasm group consists of tumors ranging from CNS WHO grade 1 to grade 3. The histological grading of ependymomas is primarily unchanged in the most recent WHO classification update, while some minor adjustments have been made. MPE tumors are the most common type of spinal cord ependymomas in adult patients and represent one methylation group, classic ependymomas are another, and spinal SE is a rare third group. An aggressive spinal cord ependymoma with early neuroaxis dissemination, anaplastic morphology, and MYCN amplification has recently been reported [6,22]. Classic ependymomas and MPEs have similar outcomes in adults, implying that the latter may be more appropriately assigned to WHO grade 2. SEs remained categorized as histological grade 1. However, MPEs were upgraded from grade 1 to grade 2 due to their clinical behavior, which includes the possibility of spread and regrowth. The current WHO classification does not provide a grade for MYCN-amplified SP-EPs. According to the WHO classification, more essential information on the fate of molecularly determined ependymoma types is required to develop a new grading system for these tumors. Ependymomas, now classified based on anatomical location and molecular sequences, have histological traits comparable to WHO grade 2 or 3. Over the last few decades, studies have produced inconclusive results regarding the relationship between histological grade and clinical success. Nonetheless, incorporating molecular classification improved the diagnostic accuracy of rare histological variants of classic ependymomas (clear cell ependymoma, papillary ependymoma, and tanycytic ependymoma), with 36% of initial diagnoses changing [19].

Location by subtype

SP-EPs are most commonly seen in the cervical spine (85.5%), followed by the thoracic spine (61.9%) and the lumbar spine (7.5%). According to research, many people have more than one ependymoma [15]. SP-MYCN develops as intramedullary or, more commonly, extramedullary large tumors with a high incidence of leptomeningeal spread in the cervical or thoracic spinal cord [23]. The conus medullaris and the filum terminale are the most common sites of SP-MPE, although rare examples of its occurrence in the brain, upper spinal cord, or even outside the CNS have been recorded [24]. SP-SE is unusual, but when it does occur, it is generally in the thoracic area [21].

Clinical features

Early symptoms are variable and may only subtly progress; their appearance before diagnosis is often in the three- to four-year range, although intratumoral hemorrhage may trigger an abrupt decline [25,26]. Symptoms and deficits are related to the specific location and extent of the tumor. Pain localized to the level of the tumor frequently occurs early in disease progression. Upper extremity symptoms predominate with cervical ependymomas. Thoracic cord tumors produce spasticity and sensory disturbances in the lower extremities [26].

Dysesthesias are the earliest manifestations to develop in 70% of patients. Numbness usually begins distally in the legs and progresses proximally. Tumors of the lumbar enlargement indicate that the tumor has thinned the surrounding spinal cord to a few millimeters; clinical findings such as back pain, extremity weakness, and bowel and bladder dysfunction indicate severe spinal cord compression [26,27].

Imaging

The preferred method for evaluating suspected spinal cord neoplasms, unless contraindicated, is magnetic resonance imaging (MRI), both with and without contrast. Due to the spinal canal's narrow width and the spinal cord's tendency to move when arterial blood flow is present, perfusion MRI and magnetic resonance spectroscopy are typically ineffective [28]. The standard sequences indicated within the assessment procedure comprise T1- and T2-weighted images in the sagittal and axial planes, followed by T1-weighted images with contrast [28].

When MRI is not an option, computed tomography (CT) should only be used to identify regions of calcification, as it has limited diagnostic utility otherwise. Ependymomas typically display hypometabolism on positron emission tomography (PET) due to their low cell density and sluggish development [13].

Ependymal Tumor Imaging Features

**Figure 2:**
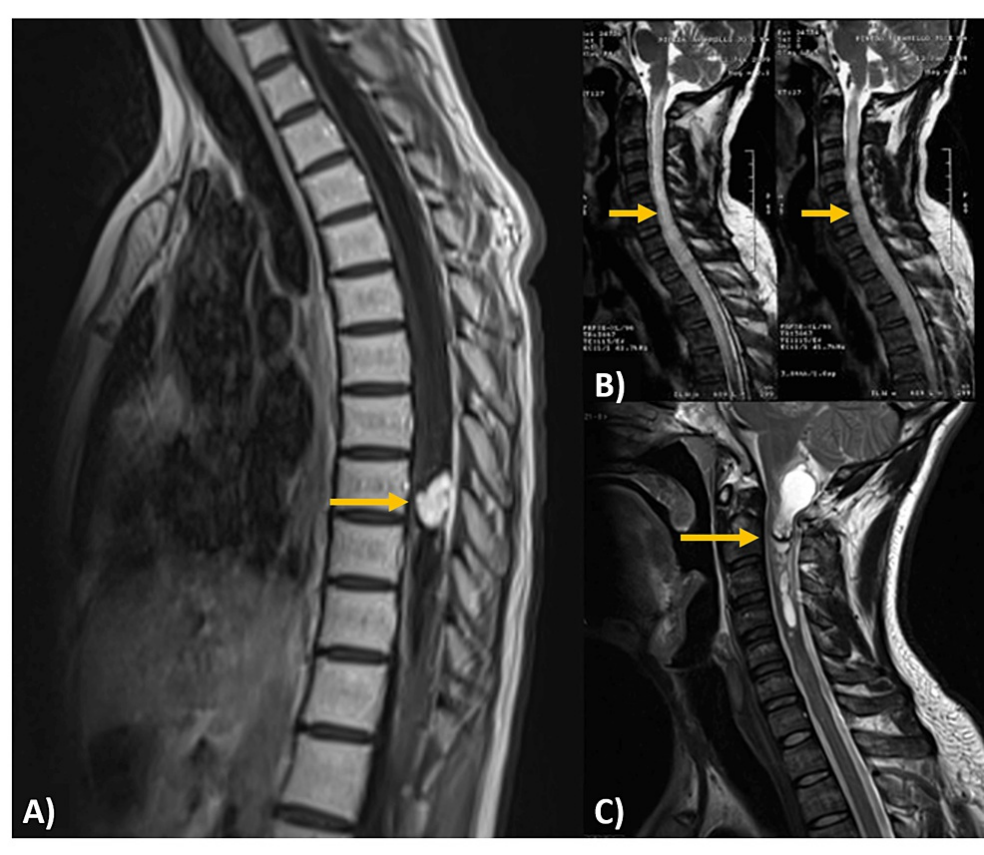
Image-based differential diagnosis (A) Magnetic resonance imaging, T1 sagittal: a thoracic intradural and intramedullary lesion with a hypervascular nodule focused on the pial surface of the spinal cord, as well as syringomyelia and medullary edema with cranial and caudal extension, and the hypervascular nodule with homogeneous and avid uptake after gadolinium administration (yellow arrow). Histological diagnosis: hemangioblastoma. (B) Magnetic resonance imaging, T2 sagittal: intradural and intramedullary cervical lesion with poorly defined margins, homogeneous and hyperintense appearance, infiltrating and expansive features (yellow arrow). Histological diagnosis: astrocytoma. (C) Magnetic resonance imaging, T2 sagittal: intradural and intramedullary cervical lesion, with solid and cystic components, leading to fusiform enlargement of the spinal cord; solid component of RM behavior (yellow arrow). Histological diagnosis: cervical ependymoma.

**Figure 3:**
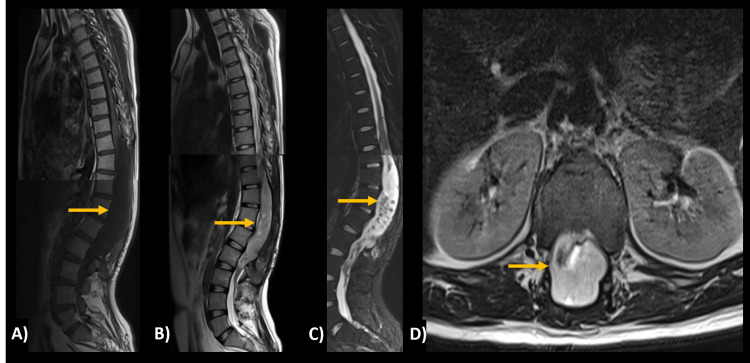
Thoraco-lumbar spinal ependymoma Intradural and intramedullary lesion with an oval shape and well-defined margins that enlarges and deforms the spinal canal at T10-L2. (A) Sagittal T1 hypointense, (B) sagittal T2 hyperintense, (C) sagittal STIR with intense and heterogeneous gadolinium enhancement and (D) axial T2 at L2 height with centripetal placement. Histological diagnosis: spinal ependymoma STIR, short tau inversion recovery

SP-EPs frequently present as a localized enlargement of the spinal cord in the cervical or upper thoracic region; indeed, they most commonly occur in the cervical region, with 44% affecting just the cervical cord (Figure 2C) and an additional 23% expanding to involve the upper thoracic cord [29]. While less common, the thoraco-lumbar location of SP-EPs accounts for a few cases (Figure 3).

They are usually concentric in position because they emanate from the ependymal-lined central canal [30]. SP-EPs are well-defined tumors, often hypointense on T1, hyperintense on T2, and contrast-enhancing concerning the spinal cord [29]. Many cases show the "cap sign," a rim of hemosiderin that manifests as a shallow T2 signal at the poles of the tumor [30].

They frequently contain cystic alteration, bleeding, necrosis, and calcification regions, which might result in a heterogeneous signal. An intramedullary cyst, typically heterogeneous rostral or caudal to the tumor, is linked to around 60% of ependymomas. Contrast enhancement can be homogeneous or heterogeneous, but it is nearly always present in some form. Although rarely found in 20% of patients, T2 hypointensity along the borders of the lesion due to distant bleeding episodes is strongly predictive of ependymoma [31].

Aside from the differences in location, the imaging characteristics of MPE are comparable to those mentioned above for ependymomas. They often show confined lesions with enhancement and a penchant for cystic and hemorrhagic alterations [32].

Unlike other ependymal tumors, SE presents as an expansive lobulated T2 hyperintense intramedullary lesion with no substantial enhancement. The bamboo leaf sign shows an eccentric or subpial development pattern on sagittal imaging, resulting in noticeable spinal cord swelling [28].

Non-ependymal Tumors: Imaging-Based Differential Diagnosis

Ependymomas are the most prevalent intramedullary spinal cord tumors, followed by astrocytomas and hemangioblastomas [29]; however, although they have similar appearances, their behavior varies considerably (Table 2, Figure 3).

**Table 2:** Image-based differential diagnosis description

Radiologic types	Ependymoma	Astrocytoma	Hemangioblastoma
Most common affected region	Cervical	Thoracic	Thoracic
Medullary location	Central canal	Eccentrically, in the cord parenchyma	Superficially, at the pial surface
Diagnostic clues	Contrast-enhanced tumors with well-defined borders that grow outward from the ependymal lining of the central canal and are usually restricted to 5 or fewer vertebral segments	A diffuse and poorly defined look that is infiltrative and lacks a definite dissecting plane	Hypervascular enhancing tumor with a cystic/syringomyelia component located on the spinal cord's pial surface
Enhancement type	Heterogeneous	No enhancement or patched	Homogeneous
Associate findings	Cystic or hemorrhagic components (cap sign), edema, syringomyelia	Cystic components, syringomyelia	Edema due to hypervascularity, syringomyelia

Astrocytomas: Astrocytomas are T1- and T2-hyperintense masses on MRI, with less cord expansion and patchy enhancement than ependymomas. They are more likely to be eccentrically positioned inside the cord and have less well-defined boundaries [31]. The average number of vertebral segments is seven [31]. Associated hemorrhage is unusual. Non-tumorous cysts are often seen. Tumoral cysts, confined inside the tumor and showing peripheral enhancement, are more frequent than ependymomas. In astrocytomas, vertebral body erosion and enlargement of the interpedicular distance are less prevalent than in ependymomas [29].

Hemangioblastomas:* *Hemangioblastomas range in size from millimeters to centimeters, are isointense to hypointense to the cord on T1-weighted imaging, and are isointense to hyperintense on T2-weighted sequences [33]. Prominent flow voids depicting dilated tortuous feeding arteries, draining veins, and peritumoral edema may be seen [32]. They are frequently associated with peritumoral cysts and syrinxes [30,33]. However, unlike ependymomas, rostral or dorsal cysts are commonly found near the cord's pial surface rather than the center [32]. On postcontrast imaging, hemangioblastomas frequently show significant homogeneous enhancement, distinguishing them from nearby peritumoral edema or non-tumorous cysts. Some instances may exhibit the typical "cyst with an enhancing nodule" look of cerebellar hemangioblastomas [30].

Pathological diagnosis

An ependymoma is a biologically diverse tumor whose molecular classification has superseded traditional histological grading based on its superior ability to characterize behavior, prognosis, and possible targeted therapies [34]. Nevertheless, establishing the diagnosis of an ependymal origin tumor requires the assessment of morphological and immunohistochemical features. In some subtypes, an aligned DNA methylation profile should be obtained; loss of chromosome 22q and amplification of the MYCN proto-oncogene should be ruled out [19].

Spinal Ependymoma

Spinal cord and intracranial ependymomas are biologically different, and there is further heterogeneity across subtypes of SP-EPs. A defining feature of SP-EPs is chromosome 22q loss, reported in 90% of cases [16].

**Figure 4:**
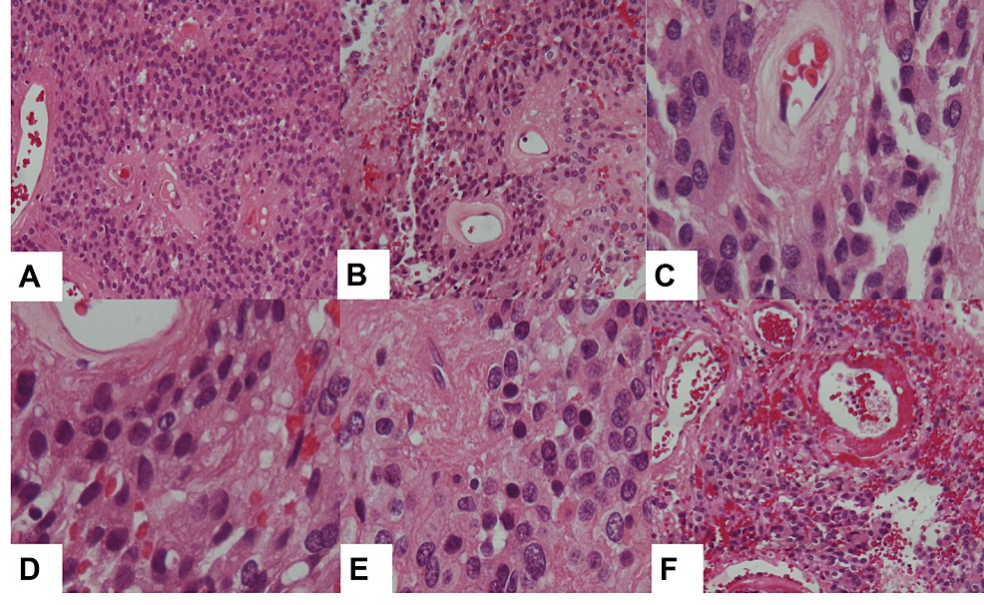
Histomorphology of ependymomas (A-F) Small hyperchromatic cells with insufficient cytoplasm produce rosette and/or pseudorosette formations surrounding vessels. (C) The cells are hyperchromatic, the vasculature has a fibrillar shape, the cells may have distinct cellular atypia, the stroma between the cells is typically fibrillar, and apparent atypia can be seen depending on the histological grade.

Macroscopically, SP-EPs are generally well-circumscribed tumors; they appear soft and mostly gray-white. They can show cystic changes, necrosis, calcification, and signs of hemorrhage [18]. The classic SP-EP form comprises monomorphic glial cells with round to oval nuclei and speckled chromatin. A characteristic feature is the pseudorosette pattern, formed by the radial arrangement of tumor cells around blood vessels, with fibrillary processes creating the perivascular anucleate zone (Figure 4). True ependymal rosettes with a central lumen are present in a minority of cases. Histological patterns, such as clear cell, papillary, or tanycytic, can be found without clinical relevance [4,13].

Immunoreactivity for glial fibrillary acidic protein (GFAP), S100 protein, and vimentin is distinctive, as is focal dot-like or ring-like intracytoplasmic immunoreactivity for epithelial membrane antigen (EMA). NHERF1/EBP50 is a protein involved in epithelial morphogenesis and is superior to EMA for diagnosing enigmatic cases [35]. In contrast to astrocytic spinal neoplasms, SP-EPs are mainly negative for OLIG2, and they do not express SOX 10, which is found in schwannoma, pilocytic astrocytoma, and most diffuse gliomas [24].

Regarding molecular assessment, SP-EPs are easily recognized and readily distinguished from other subtypes [22]. Occasionally, an SP-EP with a classic morphology exhibits the DNA methylation profile of MPE [24]. The prognostic significance of a myxopapillary DNA methylation profile in the face of an apparent discordant morphological diagnosis remains to be clarified. The NF2 gene is a tumor suppressor gene located on chromosome 22q, and mutation or deletion of either is characteristic of spinal cord ependymomas in up to 90.5% of the cases [22,36]. By definition, MYCN amplification is absent [37].

Spinal Subependymomas

A typical spinal subependymoma (SP-SE) histological section exhibits small and round-to-oval nuclei clustering in a voluminous matrix of fibrillary cytoplasmic processes [10]. Just as classic ependymomas can focally show SE-type histology, so may SEs manifest perivascular pseudorosettes. Sclerotic and ectatic blood vessels, hemorrhage, and hemosiderin deposits are common. Oddities include melanotic pigmentation [10].

SP-SEs exhibit expanded GFAP immunoreactivity and can expose focal dot-like EMA expression, but they do so less frequently than ependymomas. Some are reported to express OLIG2 or synaptophysin [10]. Also reported is the expression of HIF1a, TOP2B, MDM2, nucleolin, and phosphorylated STAT3 [17].

A determining characteristic in diagnosing this type of tumor is the deletion of chromosome 6q [22]. The two genetic analyses with more patients affected by this tumor subtype have shown a loss of 6q [22,24]. Other molecular studies have indicated reduced copies in 19p and 19q, although this assertion has not been consistent in other studies [22]. The 6q deletions were markers of good prognosis and survival [24]. TRPS1 mutations have been documented [38].

Spinal Myxopapillary Ependymomas

SP-MPE are unique in that they have an elevated expression of HIF1α, hexokinase 2 (HK2), pyruvate dehydrogenase kinase 1 (PDK1), phosphorylation of the pyruvate dehydrogenase subunit PDHA1, and decreased pyruvate kinase M activity, suggesting that a Warburg metabolic phenotype may drive this subtype. Another unique feature of this entity is an amplification of a gene encoding a tRNA thiolation protein (CTU1), which occurs in 25% of MPEs [16].

**Figure 5:**
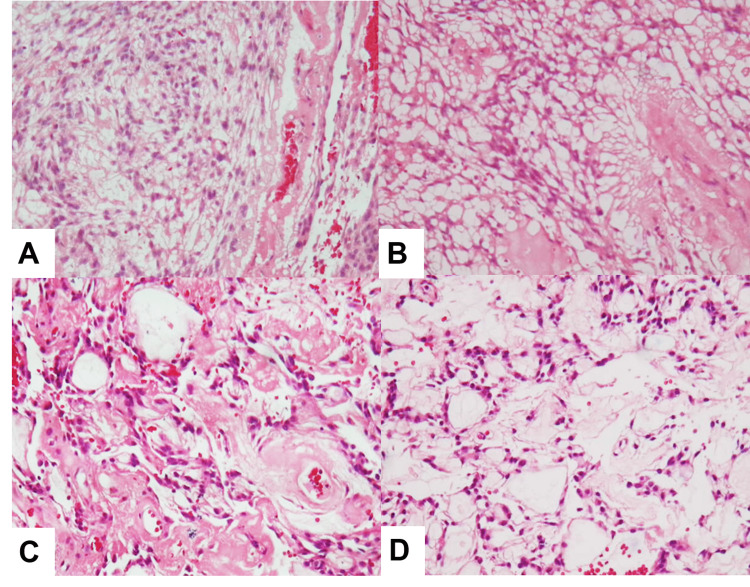
Histomorphology of myxopapillary ependymomas (A) Solid regions with flexible stroma, (B) papillary areas, (C) hyalinized vessels, (D) few cells with thicker hyalinized vessels.

Often, MPEs are soft and pink to tan-gray, may be grossly gelatinous, and can manifest cystic changes and hemorrhage [39]. Histopathologically, SP-MPEs comprise well-differentiated cuboidal to elongated tumor cells radially oriented around hyalinized fibrovascular cores, commonly with degeneration-derived myxoid accumulation (Figure 5) [39].

Diffuse immunoreactivity for GFAP distinguishes MPEs from metastatic carcinomas, paragangliomas, schwannomas, chordomas, and myxoid chondrosarcomas. Immunolabeling for S100 is also typical, and reactivity for CD99 and CD56 is frequent. MPEs are often labeled by the AE1/AE3 pan-cytokeratin cocktail, but they are generally negative for CAM5.2, CK5/6, CK7, and CK20 [40].

MPEs with a classic morphology are easily recognized, but these tumors also have a unique DNA methylation profile [22]. However, tumors with the histopathological features of classic ependymomas, particularly lumbosacral lesions with tanycytic or papillary patterns, may also cluster with MPEs. This reflects that MPEs can exhibit little myxoid change, form pseudorosettes of the usual ependymal type, and all manifest tanycytic features. Recurrent gains of chromosome 16 and losses of chromosome 10 have been documented [41].

Spinal Ependymoma MYCN-Amplification

The macroscopic features of this particular tumor subtype have not yet been described. SP-MYCN displays pseudorosettes and can have a papillary or pseudopapillary architecture [37]. Most have high-grade histopathological features, including plentiful mitotic activity, microvascular proliferation, and necrosis [42]. In immunohistochemistry, tumor cells express GFAP and show a focal cytoplasmic dot-like pattern of EMA expression [37]. MYCN protein expression can be detected by immunohistochemistry [42].

High-level MYCN amplification is present and remains stable at relapse. Additional chromosomal copy number alterations occur with a variable frequency, including loss of chromosome 10 in 32% of cases and focal losses on chromosome 11 in 26% of cases. The MYCN-amplified SP-EP has a DNA methylation profile distinct from other ependymal tumor types [23].

Treatment

Surgical tumor resection, radiotherapy, and, in certain circumstances, chemotherapy are all alternatives for treating spinal cord ependymomas [43]. Historically, surgery has been the standard treatment for spinal cord ependymomas; gross total resection (GTR) has yielded the most significant outcomes, offers the best prognosis, and should be performed at an early stage of the disease whenever possible as a functional outcome is related to small tumor size and good neurological status at the time of surgery [44].

There are few cases, such as infiltration of the spinal cord or nerve roots, in which a GTR cannot be performed with functional outcomes; when it occurs, postoperative adjuvant radiotherapy is employed [45]. During spinal surgery, intraoperative neurophysiological monitoring (IONM) is critical. It is essential to emphasize the significant impact that general anesthesia can have on IONM efficacy. Collaboration between anesthesiologists, neurophysiologists, and surgeons improves clinical outcomes after spinal surgery [46].

All patients with WHO grade 3 SP-EPs and incompletely resected WHO grade 2 SP-EPs should receive post-operative radiotherapy with 45-54 Gy [1]. Completely resected WHO grade 2 SP-EPs can be monitored without radiotherapy. A literature review on 348 patients with WHO grade 2 and 3 SP-EPs has shown that radiotherapy prolonged the progression-free survival (PFS) in patients receiving subtotal resection (STR). The median PFS was 48 months in patients treated with STR alone and 96 months for patients treated with STR followed by radiotherapy [45,47].

Chemotherapy may be warranted for patients with recurrent ependymomas who are no longer eligible for local treatments [45]. In adults, chemotherapeutic agents such as temozolomide can be considered, while in children, the choice of chemotherapeutic drugs depends on previous exposures [45,48].

Temozolomide, a chemotherapeutic agent that is used in treatment regimens for adult glioblastoma, has also been studied in adult patients with ependymomas. A 2021 trial of dose-dense temozolomide and daily lapatinib, a tyrosine kinase inhibitor, in 50 adult patients with recurrent supratentorial, infratentorial, and spinal cord ependymomas demonstrated prolonged disease control, with 6-month and 12-month PFS rates of 55% and 38%, respectively; this treatment combination may thus be considered as an option for salvage therapy in adult patients with recurrent ependymomas [49].

Prognosis

Although age at diagnosis, sex, race, treatment modality, histological subtype, extent of resection, location, and size of the tumor are influential variables, SP-EPs are associated with a favorable outcome in children and adults, with progression-free survival and overall survival (OS) rates of 70%-90% and 90%-100%, respectively, over 5-10 years [10]. However, PFS declines over time, reflecting many late relapses [50]. The extent of resection is a prognostic factor in most studies, with patients with gross total resection having favorable PFS [50].

PFS and OS by subtype

Despite the risk of recurrences and SP-EP development, overall survival is excellent [22]. SP-MYCN is characterized by early metastases, fast progression upon relapse, leptomeningeal spread, and poor response to multimodal therapy methods [23]. A case series of 13 tumors revealed a high recurrence rate of 75%-100% with a median PFS of 17 months [37]. In this cohort, the median OS was 87 months. High-level MYCN amplifications are seen in all cancers and remain stable upon relapse [23].

Patients with SP-MP have a favorable clinical prognosis, with long-term OS rates surpassing 90% after whole or partial surgical resection [51]. However, relapses occur in around 20% of MPE patients, locally or at a distant location. Recurrence is linked to several clinical factors, including subtotal resection, tumor size, and multifocal lesions [52]. Overall, the SE prognosis is favorable, and recurrence, even after subtotal resection, is rare [22].

## Conclusions

This review signifies remarkable progress in our understanding of neuroepithelial tumors, particularly in the context of spinal cord ependymomas, known for their complexity even among experienced surgeons. Aligned with the World Health Organization's classification system, the integration of molecular techniques offers the potential for precise treatment strategies, bolstering patient care. Its exhaustive review, placing these tumors within the 2021 WHO Classification of Tumors of the Central Nervous System, sets a standard for meticulous inquiry.

This work guides researchers, clinicians, and scholars, shedding light on new avenues for diagnosis and treatment. Its impact resonates beyond its pages, deepening comprehension of ependymomas, advancing diagnostics, and catalyzing innovative treatments. As medicine advances, this study's insights promise to shape spinal ependymoma management, inspiring optimism and excellence in patient care. Ultimately, these advancements can potentially improve treatment outcomes for individuals with spinal ependymomas, offering a more tailored and effective approach in the clinical setting.
